# Standardized Duplex Ultrasound-Based Protocol for Early Diagnosis of Transplant Renal Artery Stenosis: Results of a Single-Institution Retrospective Cohort Study

**DOI:** 10.1155/2018/2580181

**Published:** 2018-04-11

**Authors:** Vincenzo Li Marzi, Riccardo Campi, Francesco Sessa, Alessandro Pili, Graziano Vignolini, Mauro Gacci, Michele Marzocco, Eugenio Dattolo, Enrico Minetti, Mariella Santini, Massimo Gatti, Adriano Peris, Sergio Serni

**Affiliations:** ^1^Department of Urological Robotic Surgery and Renal Transplantation, University of Florence, Careggi Hospital, Florence, Italy; ^2^Department of Nephrology, University of Florence, Careggi Hospital, Florence, Italy; ^3^Department of Cardiology and Angiology, University of Florence, Careggi Hospital, Florence, Italy; ^4^Intensive Care Unit and Regional ECMO Referral Centre, Azienda Ospedaliero-Universitaria Careggi, Florence, Italy

## Abstract

Transplant renal artery stenosis (TRAS) is the most frequent vascular complication after kidney transplantation (KT) and has been associated with potentially reversible refractory hypertension, graft dysfunction, and reduced patient survival. The aim of the study is to describe the outcomes of a standardized Duplex Ultrasound- (DU-) based screening protocol for early diagnosis of TRAS and for selection of patients potentially requiring endovascular intervention. We retrospectively reviewed our prospectively collected database of KT from January 1998 to select patients diagnosed with TRAS. The follow-up protocol was based on a risk-adapted, dynamic subdivision of eligible KT patients in different risk categories (RC) with different protocol strategies (PS). Of 598 patients included in the study, 52 (9%) patients had hemodynamically significant TRAS and underwent percutaneous angioplasty (PTA) and stent placement. Technical and clinical success rates were 97% and 90%, respectively. 7 cases of restenosis were recorded at follow-up and treated with re-PTA plus stenting. Both DU imaging and clinical parameters improved after stent placement. Prospective high-quality studies are needed to test the efficacy and safety of our protocol in larger series. Accurate trial design and standardized reporting of patient outcomes will be key to address the current clinical needs.

## 1. Introduction

Kidney transplantation (KT) represents the treatment of choice for end-stage renal disease (ESRD) [1]. Although both outcomes and survival have been improved by standardization of surgical techniques, perioperative management, and immunosuppressive regimens, postoperative complications remain frequent in surgical practice [2]

Transplant renal artery stenosis (TRAS) is considered the most frequent vascular complication after KT with an overall incidence of 1–23% and prevalence of 1,5–4% [1]. TRAS is generally observed 3–24 months after KT [3] and its incidence has progressively increased due to a more extensive use of noninvasive diagnostic procedures [4]. However, to date, conventional angiography remains the gold standard for diagnosis and provides the guidance for percutaneous transluminal angioplasty (PTA) with or without endovascular stenting [1].

As TRAS has been associated with potentially reversible refractory hypertension [5], graft dysfunction, and reduced patient survival [6, 7], its early diagnosis and prompt management still represent key clinical needs.

Nonetheless, the wide variability of reported incidence rates of TRAS may reflect the lack of standardized definitions of hemodynamically significant disease [8], clinical relevance [1], and technical/clinical success in current surgical series [4]. Of note, the lack of specific Doppler ultrasound follow-up protocols prevents a prompt diagnosis of asymptomatic TRAS in most patients, with potential delay of treatment and associated worsening of long-term graft renal function. Therefore, a critical unmet need is to select a noninvasive diagnostic work-up to screen asymptomatic KT patients to early diagnose those patients with hemodynamically significant TRAS deserving further invasive diagnostic and therapeutic procedures [4].

The aim of the study is to describe the outcomes of our standardized screening protocol in patients undergoing KT focusing on the role of Duplex Ultrasound (DU) imaging for early diagnosis of TRAS and for selection of patients potentially requiring endovascular intervention.

## 2. Materials and Methods

### 2.1. Surgical Technique

All KTs were performed with open surgery by a dedicated transplant team composed by 5 highly experienced surgeons according to a standard operative technique [9]. For KT, end-to-side anastomoses were performed between the graft vessels and the external iliac vein and artery using a Carrel patch. In two cases, an end-to-end arterial anastomosis using the internal iliac artery and, in 13 cases, an end-to-side arterial anastomosis between the graft renal artery and common iliac artery were performed. Ureteral neocystostomy was performed extravesically according to the Lich-Gregoir or Barry techniques using a double J ureteral stent as guidance.

### 2.2. Patient Population and Study Design

After Institutional Ethical Committee approval, our prospectively collected database of patients undergoing KT from brain-dead* heart-beating* donors at Careggi University Hospital from January 1998 was retrospectively reviewed to select patients enrolled in our standardized DU-based follow-up protocol diagnosed with TRAS.

### 2.3. Duplex Ultrasound Criteria for Suspicion of TRAS

The evaluation of the vascular axis of the transplanted kidney was performed according to standardized imaging protocols [8]. All DU evaluations were performed by 4 radiologists with extensive experience in KT imaging.

DU criteria for suspicion of TRAS used in our study are depicted in Table 1. In particular, DU imaging was focused on the research of pathological vascular flows within the arterial axis of the transplanted kidney that were suspect for hemodynamically significant stenosis.

No specific morphological abnormalities of the transplant renal artery were considered sufficient to raise the suspicion of TRAS, independently from their location (i.e., anastomotic and postanastomotic).

In our protocol, an increase in systolic peak velocity (SPV) at the level of the flow abnormality at DU imaging (site of the potential stenosis) was considered the mandatory parameter to raise the suspicion of TRAS. Other DU parameters traditionally associated with TRUS were (a) the manifestation of* tardus-parvus* waveform and (b) reduced resistive indexes (RI) at parenchymal level. However, being affected also by parenchymal conditions, they represent indirect signs not specific for TRAS not sufficient nor necessary for DU suspicion of TRAS.

### 2.4. Follow-Up Protocol

The DU-based follow-up protocol for early detection of TRAS was performed in addition to the regular follow-up visits routinely defined by the KT Protocol at our institution in accordance with the current international Guidelines [10]. Estimated glomerular filtration rate (eGFR) was calculated using the CKD-EPI 2009 formula and was used as a surrogate for allograft function, while systolic, diastolic, and mean arterial blood pressure (ABP) was used to assess cardiovascular status.

According to our protocol, DU imaging was scheduled in all eligible patients at day 3 after KT, at discharge, then at 1, 3, 6, and 12 months, and annually thereafter.

The timing of DU investigations was defined according to the standard surveillance protocols used in vascular surgery after carotid stenosis treatment [11].

The follow-up protocol was based on a risk-adapted, dynamic subdivision of eligible KT patients in different risk categories (RC) according to (a) presence of symptoms, namely, refractory hypertension, that is, failure to achieve optimal blood pressure control to levels less than 140/90 mm Hg despite the concomitant use of 3 or more different classes of antihypertensive agents and/or worsening of renal function (rising of serum creatinine >20% of basal value, after excluding all other potential sources of graft impairment) [12]; (b) DU criteria for suspicion of TRAS (Table 1).

*Asymptomatic* patients were assigned to RC 1, RC 2, or RC 3 if DU imaging was negative for suspicion of TRAS (VPS < 2,2 m/sec at the level of presumed arterial stenosis), suspicious for TRAS (VPS > 2,2 m/sec but lower than 2,8 m/sec), or suspicious for hemodynamically significant TRAS (VPS > 2,8 m/sec), respectively. On the contrary,* symptomatic* patients were classified into RC 4 and RC 5 based on DU findings as follows: RC 4, if VPS < 2,2 m/sec; RC 5, if VPS > 2,2 m/sec, in both cases after differential diagnosis excluded other potential causes of refractory hypertension or worsening renal function.

Our approach ultimately identified three different protocol strategies (PS): PS1, continuation of regular follow-up imaging (at the predefined time intervals); PS2, intensification of the follow-up schedule (DU imaging monthly until reclassification in a different RC within 1-year period); PS3, indication for conventional angiography and possible concomitant PTA treatment, after a confirmatory DU examination.

According to the above discussed principles, each KT patient was assigned a RC and a specific PS as follows (Table 2):

- RC 1 patients continued PS1.
- RC 2 and RC 4 patients were followed more strictly (PS2) in order to be dynamically reclassified within an established period (1 year) in a different RC.
- RC 3 and RC 5 patients were candidates for immediate angiography +/− PTA and stenting.

### 2.5. Angiography and Endovascular Intervention

In patients with high suspicion of TRAS (RC 3 and RC 5), conventional or digital subtraction angiography was performed according to the standard technical principles [13].

According to the results of angiographic imaging, patients were reclassified into the following groups:

1. Patients with no angiographic evidence of TRAS
2. Patients with angiographic evidence of TRAS < 50%
3. Patients with angiographic evidence of TRAS > 50% and <70%
4. Patients with angiographic evidence of TRAS > 70% (hemodynamically significant TRAS).

**Figure 1:**
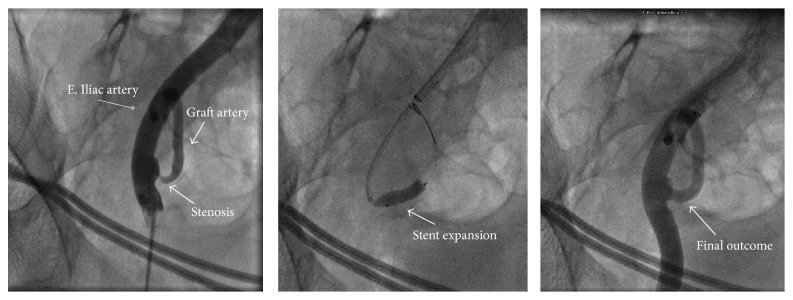
*Percutaneous angioplasty and stent placement for anastomotic TRAS*. In all patients, a nonselective aortoiliac arteriogram was performed to exclude any preanastomotic (proximal-TRAS) inflow stenosis in the recipient arteries. The stenotic lesion was dilated with a 4.8-French angioplasty balloon catheter passed through a valved 8-French introducer sheath via a femoral approach. Where indicated, the intravascular stent (Palmaz endoprosthesis) was implanted over a stiff 0.5 mm guide wire passed carefully through the stenosis, measuring the pressure gradients. In case of hemodynamically significant stenosis, 3000–5000 U of heparin was administered intravenously and balloon dilation is performed. Balloon-size selection was based on direct measurement of the diameter of a normal nondiseased renal artery segment, as previously described (hederman). Then the bare-metal stent was inserted and left in place in the transplant renal artery after the removal of the guide wire and balloon catheter. A postangioplasty arteriogram was always obtained.

Techniques of angiography, PTA, and stent placement were previously described [14] and shown in Figure 1. Patients with angiographic evidence of TRAS > 70% were always treated with PTA and stenting if hemodynamic pressure measurements showed a systolic pressure gradient of 20 mmHg or more. On the contrary, patients with TRAS of 50–70% were treated with PTA only if the systolic pressure gradient did not exceed the threshold of 20 mmHg.

*Technical* success was defined as an endovascular intervention resulting in complete restoration of renal allograft perfusion without any significant residual stenosis as determined by a negligible systolic pressure gradient or fluoroscopic visualization in cases where pressure measurements were unavailable. In particular, technical success was achieved in case of residual stenosis less than 30% after endovascular intervention, with residual peak systolic pressure gradient less than 10 mmHg across the lesion. After endovascular intervention, all patients continued the follow-up according to the PS1 schedule. For asymptomatic patients with TRAS treated with PTA and stenting, definition of* clinical* success relied on the absence of restenosis of transplant renal artery during the long-term follow-up requiring reintervention (either repeated PTA +/− stent or open surgery). For symptomatic patients with TRAS treated with PTA and stenting, beyond the previously described criterion, clinical success was also defined as reduction of ABP values and of the number of chronic antihypertensive medications, as well as improvement/stability in renal function.

Both clinical (systolic and diastolic blood pressure, eGFR) and DU parameters (PSV, RI) of patients undergoing PTA + stenting were recorded at 1-month follow-up after the endovascular procedure.

### 2.6. Allograft Survival

Allograft loss was defined by the need for permanent dialysis as documented by the renal transplant team notes, which occurred at regular intervals following KT. Outcomes in allograft survival were censored for patient survival.

### 2.7. Statistical Analysis

Statistical analyses and reporting of results were conducted according to recently published [15]. First, descriptive statistics were obtained for all variables. Since sample data failed to meet most assumptions for parametric testing, all statistical analyses were performed with nonparametric tests. Continuous variables are presented as medians and interquartile (IQR) range, while categorical variables are presented with frequencies and proportions. The Wilcoxon signed-rank test was used to compare mean SPV, RI, Systolic ABP, diastolic ABP, and eGFR values among the TRAS-patients before and after stent placement. The Kruskal-Wallis test was used to compare the mean ΔSPV, ΔRI, ΔSBP, ΔDBP, and ΔeGFR values among the TRAS-patients at different time periods from renal transplantation (<3 months, 3–12 months, and >12 months). All tests were two-sided with a significance level set at *p* < 0.05. All statistical tests were performed using SPSS v.18.0 (IBM Corp., Armonk, NY, USA).

## 3. Results and Discussion

### 3.1. Results

Overall, 946 patients underwent KT at our institution from July 1991. Of these, patients undergoing KT before January 1998 (140/946, 15%) were not eligible for the study as there was no standardized follow-up protocol for early detection of TRAS.

Of the 806 patients undergoing KT from January 1998, patients experiencing medical or surgical complications in the postoperative period, patients with unavailable clinical data, and patients lost at follow-up were excluded from the study.

Thus, 620/806 (77%) were enrolled within a standardized, DU-based follow-up protocol for early detection of TRAS.

During follow-up, 18/620 (3%) patients for whom serial DU imaging could identify the presence of morphological transplant renal artery kinking (TRAK) were also excluded from the study protocol.

Finally, 598 patients had complete data available and constituted our study population.

The flow-chart detailing the study design is shown in Table 3.

Of 598 KT patients included in the study, 59 (10%) patients with clinical or DU suspicion of TRAS underwent diagnostic angiography and 56/59 (95%) patients were diagnosed with TRAS at angiographic evaluation. Thus, the incidence of TRAS in our series was 9%.

Of patients diagnosed with TRAS, 35/56 (62%) were male and 21/56 (38%) female. Median age at KT was 55 years (IQR 43–69). Median time from KT to TRAS diagnosis was 71 days (IQR 22–130). Most of the TRAS were located at the level of the anastomosis (30/56, 54%), while the other cases were preanastomotic (6/56, 10%) or postanastomotic (20/56, 36%).

In 3/59 (5%), no evidence of TRAS was shown, of which there was 1 case of TRAK. These patients continued the regular noninvasive follow-up protocol (PS1).

Among the 56 patients with angiographic diagnosis of TRAS, 1 case showed a TRAS < 50% and no further treatment was performed (PS1). In 3 (5%) patients, a TRAS of 50–70% with a peak systolic pressure gradient across the stenosis of <20 mmHg was shown; in these cases, PTA was performed without stent placement.

In 52/56 (93%) patients hemodynamically significant TRAS with a peak systolic pressure gradient > 20 mmHg was shown and underwent PTA plus bare-metal stent placement. No major complications were recorded after endovascular intervention. There were two minor hematomas which were managed conservatively.

Overall technical success rate was 97%, while clinical success rate was 90%.

During follow-up, 8/56 (14%) patients fulfilled the criteria for a new angiographic evaluation (i.e., new onset of symptoms or worsening of renal function with DU imaging suspicious for TRAS).

Overall, 7 cases of restenosis were recorded at a median time from first diagnosis of 16 months (IQR 4–20) and were treated with a bare-metal stent (6 restenting procedure and 1 de novo procedure in a patient previously treated with PTA). In one case, digital angiography did not show hemodynamically significant TRAS.

Overall, of the 52 patients treated with PTA + stent placement, 18/52 (34%) patients died after a median (IQR) survival of 87 (49–130) months and 9/52 (18%) were lost to follow-up (Supplementary Figure  1). Causes of death included neoplastic diseases, cardiovascular/respiratory events or infections (Supplementary Table  1). Median (IQR) graft survival was 87 (49–130) and 58 (56–119) among patients that died with (*n* = 15) and without (*n* = 3) a functioning graft, respectively. On the contrary, among the 25 alive patients, after a median (IQR) follow-up of 154 (79–176) months, 18/25 (72%) have currently a functioning graft while 7/25 (28%) require redialysis. Median (IQR) graft survival among these patient cohorts was 128 (73–155) and 113 (85–130) months, respectively (Supplementary Figure  1).

Table 4 shows the comparison of mean SPV, RI, Systolic and diastolic ABP, and eGFR values among the 52 TRAS-patients before and after stenting placement, while Table 5 describes the comparison of the results of stent placement in patients treated at different time periods from KT (first 3 months, 3–9 months, and 1 year of after).

Overall, both DU imaging and clinical parameters improved after stent placement. In particular, median value of mean SPV at the level of TRAS was significantly lower after stent placement (1,4 versus 3,0 m/sec, *p* < 0,001), as well as diastolic blood pressure (80 versus 85 mmHg, *p* = 0,06). Median values of mean RI were significantly higher after the procedure (0,72 versus 0,68, *p* = 0,01); both systolic blood pressure and serum creatinine values were also improved after endovascular intervention, even if not reaching statistical significance (Table 4).

There was no statically significant difference between the single Δ (post − pre) values of PSV, RI, systolic or diastolic ABP, and eGFR among patients with TRAS treated at different time periods from KT (Table 5).

### 3.2. Discussion

Our study aimed to provide evidence on the outcomes of a standardized DU-based follow-up protocol of patients undergoing KT for screening and early diagnosis of TRAS through a retrospective analysis of a large series. We found an overall incidence of TRAS of 9% (56/598 patients, Table 3). The technical and clinical success rates of PTA and stenting for patients with hemodynamically significant TRAS were 97% and 90%, respectively.

Several studies have highlighted the negative prognostic role of TRAS in terms of allograft dysfunction, refractory hypertension, and inferior graft survival in absence of prompt intervention [6, 16, 17]. Unfortunately, the actual incidence of TRAS is still unclear in the KT literature with broad ranges (1–23%, [17]) due to the lack of standard definitions of clinical or DU diagnostic criteria for the disease [1, 4]. At the same time, TRAS might have a multifactorial etiology that may generate confusion regarding its best management [1].

In this scenario, early diagnosis of TRAS is key to improve the care of KT patients by selecting those deserving invasive therapeutic procedures. However, to date, diagnosis of TRAS still relies on angiographic imaging, exposing many patients to invasive, costly, and potentially harmful diagnostic techniques. In this regard, the number of patients with suspicion of TRAS that needs to undergo conventional angiography to detect a hemodynamically significant disease is still unclear [10], raising concerns of the true utility of angiography as the gold standard diagnostic tool. Moreover, computed tomography angiography and magnetic resonance angiogram are still no ideal diagnostic techniques, as they are costly, not easily available, potentially associated with graft toxicity and unable to provide guidance for concomitant treatment. Therefore, the most recent Guidelines of the European Association of Urology (EAU) on KT stress the concept of DU imaging as a noninvasive, economical screening technique to select patients with suspicion of TRAS requiring further diagnostic and therapeutic interventions. Yet, no standardized follow-up protocols based on DU are proposed [10]. In this context, our study addresses the critical unmet need of defining a standardized, noninvasive diagnostic work-up to screen* asymptomatic* KT patients for TRAS to select those deserving further invasive diagnostic and therapeutic procedures. Our standardized follow-up protocol was designed to adjust the type and intensity of follow-up investigations according to the individual patient's risk of hemodynamically significant TRAS based on DU criteria (Table 1). The objective of such protocol was indeed to early detect all cases of hemodynamically significant TRAS that would eventually require conventional angiography, with or without PTA and stenting. To achieve the goal, in the context of extreme variability of DU diagnostic criteria of TRAS reported in the current series [4], we defined a simple and reproducible DU predictor of suspected TRAS, namely, SPV at the level of the presumed stenosis at DU imaging. Being directly influenced by the degree of flow abnormalities rather than indirect signs of parenchymal damage, SPV potentially overcomes the traditional DU criteria associated with TRAS (tardus-parvus waveform and reduced RIs) that we consequently considered only accessory criteria for diagnosis of suspected TRAS.

The rational for our standardized follow-up protocol based on regular DU investigations is to arouse suspicion of TRAS even in nonsymptomatic cases. To improve further the diagnostic performance of the DU technique, we defined different categories of risk (RC) at the individual patient level by merging clinical and DU parameters (Table 1) to select proper risk-adjusted protocol strategies. As such, (1) asymptomatic patients required higher SPV values than those of symptomatic patients to be eligible for conventional angiography; (2) in each category (asymptomatic versus symptomatic), SPV values acted as drivers to select the most suitable PS (i.e., in asymptomatic patients, PS 1 if SPV < 2,2 m/sec and PS 3 if SPV > 2,8 m/sec). With such classification system patients could also be dynamically reclassified into specific RCs in case of borderline SPV values in both symptomatic and asymptomatic patients by following a more strict follow-up schedule.

Overall, our protocol defined two distinct operative follow-up strategies, of which one is conservative (PS1, that is, regular follow-up at predefined time intervals, Table 2) and one is interventional (PS3, namely, the indication for further invasive procedures). In addition, to avoid useless angiographic procedures in those patients with unclear RC, our protocol considered a specific protocol strategy (PS2) that aimed to reclassify patients into PS1 or PS3 based on the results of serial DU investigations.

The theoretical principles of the protocol were confirmed by our results.

In our series, 52 patients were diagnosed with TRAS at angiographic imaging and underwent PTA and stent placement. Overall, the values of systolic and diastolic ABP, as well as eGFR, were improved after stent placement, although they did not reach statistical significance (Table 4). However, the absolute values of systolic ABP before treatment were lower than previously reported [18, 19], potentially reflecting the ability of our protocol to raise suspicion of TRAS in more asymptomatic patients with lower values of ABP and, consequently, to achieve earlier diagnosis and treatment of the disease. On the contrary, SPV values were significantly higher before stent placement (3,0 m/sec versus 1,4 m/sec). This finding underlines the ability of SPV to detect early abnormal flow variations at the level of stenosis in patients undergoing DU follow-up imaging and to act as surrogate of the treatment efficacy.

Regarding the functional outcomes after endovascular intervention, our study failed to show a statistically significant improvement in graft renal function after the procedure, as described by previous series [17, 20, 21]. This finding might have different explanations. First, early diagnosis of TRAS achieved by our protocol, especially in asymptomatic patients, may have prevented patients to develop a relevant functional graft impairment before the procedure. However, the heterogeneity of the cohort might have dimmed a true functional benefit of the endovascular intervention. In particular, the lack of stratification of patients undergoing PTA + stenting in the specific RC 3 and RC 5 (namely, asymptomatic patients with VPS > 2,8 m/sec and symptomatic with VPS > 2,2 m/sec, resp., Table 2) did not allow us to compare pre- and postfunctional outcomes of endovascular intervention separately for the two risk groups and then to detect a potential overtreatment of selected patients with TRAS.

Finally, the potential delay of diagnosis associated with our protocol may have hindered the possibility of renal function improvement of TRAS treatment. Of note, mean eGFR values at the time of treatment showed a relative stability of graft renal function, reducing the likelihood of a negative effect of time to TRAS diagnosis on functional outcomes.

We also tested the hypothesis that the time from KT to TRAS diagnosis might influence the outcomes of endovascular procedure (Table 5). The absence of statistically significant differences between the Δ values of both ABP, SPV, and eGFR among patients diagnosed with TRAS <3 months, 3–12 months, and >12 months after KT might reflect a relative independence of treatment's results from the time to TRAS diagnosis. Thus, our follow-up protocol does not seem to delay the time for TRAS diagnosis and treatment nor to worsen the final functional results of endovascular treatment. Moreover, as confirmed by several reports [1, 10], most TRAS occurred after 12 months from KT, being potentially related more to the individual biological characteristics of the recipient rather than technical factors of KT.

Major strengths of our study are

- the use of a standardized follow-up protocol for screening and early diagnosis of TRAS in a large surgical cohort based on a noninvasive, low-cost diagnostic modality (DU) defining specific patient RCs and risk-adapted dynamic PSs;
- the use of SPV as a reliable, reproducible and efficient DU parameter to screen, monitor and select patients with suspicion of TRAS for different protocol strategies;
- the application of a well-defined follow-up protocol in both symptomatic and asymptomatic patients to improve the rate of early TRAS diagnosis;
- the high rates of both technical and clinical success of endovascular intervention in patients with diagnosis of hemodynamically significant TRAS selected by the protocol.

Besides its strengths, our study is not devoid of limitations. First, this is a retrospective study of a large patient cohort covering a rather long study period. Therefore, the study might be prone to selection, detection, and attrition biases. In this regard, we were not able to subcategorize patients with angiographic diagnosis of TRAS undergoing stent placement within the RC 3 and RC 5 categories. As such, we could not perform comparative statistical analyses between RC 3 and RC 5 patient categories to detect differences among these groups regarding the functional benefit of endovascular intervention. This lack of specific information on RC patient category might have represented a relevant* limitation* of the study, especially for the interpretation of the functional outcomes. At the same time, this represents a significant starting point for future studies based on our follow-up protocol. It is also important to consider that, to date, there is no evidence on the potential clinical benefit (or disadvantage) of* early *versus* delayed *treatment of TRAS in asymptomatic patients. Therefore, we designed our DU-based protocol with the aim of diagnosing* early* significant TRAS after KT and treat it, if confirmed by angiographic imaging, as soon as possible to minimize the future potential detrimental consequences of TRAS.

Second, our findings might not be completely generalizable outside tertiary referral centres as both DU investigations and KTs were performed by highly experienced teams.

Third, due to its inherent characteristics, our protocol cannot provide reliable estimates of the false-negative rate of patients with TRAS (i.e., % of asymptomatic patients with DU parameters negative for suspicion of TRUS that actually had TRAS at angiographic imaging). However, this patient category is rare in current clinical practice and difficult to detect with conventional diagnostic criteria. Both the DU criteria for suspicion of TRAS and the specific RCs and PSs used in our protocol were adapted from available definitions in the literature and defined according to our personal experience. However, as shown in a recent systematic review of the literature, there is wide heterogeneity regarding the definitions of TRAS diagnostic criteria, the triggers for interventions, the reporting of treatment outcomes, and types of follow-up schedules [4, 21, 22]. As such, we designed a follow-up protocol that could capture the different risk of TRAS in the single KT patient and adjust the intensity and invasiveness of treatment accordingly. Finally, our data were insufficient to provide evidence on the potential need of a more intensive DU-based follow-up schedule in specific patient categories (i.e., diabetic, atherosclerotic aortoiliac disease, and recipients of marginal donors).

Despite these limitations, our findings afford opportunities for significant further research. Prospective high-quality studies are needed to (a) test the efficacy and safety of our protocol in larger series; (b) evaluate the diagnostic accuracy of such protocol compared to the current gold standard approach based on conventional angiography for diagnosis of TRAS; and (c) evaluate the potential clinical advantages of personalized DU-based follow-up schedules for early diagnosis of TRAS in patients at higher risk of TRAS and TRAS-related complications. To do this, accurate trials design and standardized reporting of patient outcomes will be key to address the current clinical needs [23].

## 4. Conclusions

TRAS represents the main vascular complication after KT. Early diagnosis and prompt management are key for patient outcomes.

We have shown the feasibility, efficacy, and safety of a standardized, risk-adjusted, DU-based follow-up protocol for screening and early diagnosis of TRAS in a large cohort of patients undergoing KT at our institution. SPV was used as the main DU parameter to raise suspicion of TRAS. Combining patient symptoms and DU findings into specific RCs allowed addressing each patient toward an appropriate PS in order to avoid unnecessary angiographic investigations and to select patients deserving endovascular interventions.

Technical and clinical success rates in patients with hemodynamically significant TRAS treated with endovascular intervention were noteworthy.

Future high-quality studies are needed to prove the efficacy of our protocol in larger series and to compare it with the current gold standard diagnostic modalities.

## Figures and Tables

**Table 1:** * Duplex Ultrasound (DU) criteria for suspicion of TRAS in the study*. SPV > 2,2 m/sec was considered the landmark value for suspicion of TRAS > 50%, while a SPV > 2,8 m/sec was considered the landmark value for suspicion of TRAS > 70%, which we considered hemodynamically significant according to the available evidence (Ngo). Tardus-parvus waveform and reduced RI were considered accessory parameters that might increase the degree of suspicion in case of symptoms or altered SPV. TRAS = transplant renal artery stenosis; SPV = systolic peak velocity; RI = resistive indexes.

*Direct criteria* *(mandatory criterion)*	*SPV > 2,8 m/sec* (suspicion of TRAS > 70%) (at the level of presumed stenosis)
	*SPV > 2,2 m/sec* (suspicion of TRAS > 50%) (at the level of presumed stenosis)

*Indirect criteria* *(accessory criteria)*	*Tardus-parvus waveform* (at parenchymal level)
	*Reduce RIs (<0,8)* (at parenchymal level)

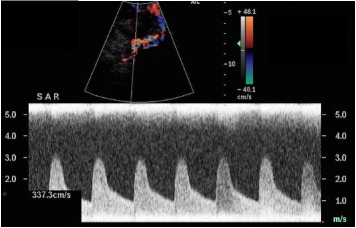	

**Table 2:** *Overview of the protocol strategies (PS) for early detection of TRAS according to the patient's individual risk category (RC)*. Patients were defined as symptomatic in case of refractory hypertension (defined as failure to achieve optimal blood pressure control to levels less than 140/90 mm Hg despite the concomitant use of 3 or more different classes of antihypertensive agents) and/or worsening of renal function (defined as rising of serum creatinine >20% of basal value, after excluding all other potential sources of graft impairment). RC1 patients continued PS1, RC2 and RC4 patients followed a stricter follow-up (PS2) to reclassify the patient in a different RC; finally, RC3 and RC 5 patients were candidate for immediate angiography +/− PTA and stenting. TRAS = transplant renal artery stenosis; SPV = systolic peak velocity.

Symptoms (refractory hypertension and/or worsening of renal function)	VPS (m/sec)	Risk category (RC)	Protocol strategy (PS)
No	<2,2	1	1. Regular ECD follow-up at 3° POD, discharge, 1, 3, 6, 12 months then annually
	2,2–2,8	2	2. ECD imaging monthly until reclassification in a different RC within 1-year period
	>2,8	3	3. Indication for angiography +/− PTA +/− stenting

Yes	<2,2	4	2. ECD imaging monthly until reclassification in a different RC within 1-year period
	>2,2	5	3. Indication for angiography +/− PTA +/− stenting

**Table 3:** Flow-chart detailing the study design. TRAS = transplant renal artery stenosis.

*Patients undergoing kidney transplantation from July 1991 at Careggi University Hospital* (*n* = 946)	
*Patients eligible for the standardized ECD- based follow-up protocol for early detection of TRAS (started on January 1998)* (*n* = 806, 85%)	*Patients undergoing KT from 1991 to 1998 excluded due to lack of standardized follow- up protocols for diagnosis of TRAS* (*n* = 140, 15%)

*Patients enrolled in the standardized follow-up protocol with complete clinical data available* (*n* = 620, 77%)	*Patient excluded due to* the following (i) Any medical or surgical complications in the postoperative period (ii) Unavailable clinical data (iii) Lack of follow-up data (*n* = 186, 23%)

*Patients enrolled in the study undergoing regular follow-up at our Institution* (Study population) (*n* = 598, 97%)	*Patients with ECD diagnosis of transplant renal artery kinking (TRAK) excluded from the study* (*n* = 18, 3%)

*Patients with clinical or ECD suspicion of TRAS undergoing diagnostic angiography* (*n* = 59, 10%)	*Patients continuing the Protocol Strategies according to the specific risk categories* (Table 2) (*n* = 539, 90%)

*Patients diagnosed with TRAS* (*n* = 56, 95%)	*Patients with no evidence of TRAS, of which 1 TRAK, continuing the regular follow-up protocol* (*n* = 3, 5%)

*Patients with TRAS < 50% continuing regular follow-up protocol* (*n* = 1; 2%)	
*Patients with TRAS of 50–70% with a peak systolic pressure gradient across the stenosis of <20 mmHg undergoing PTA only* (*n* = 3; 5%)	
*Patients with TRAS > 70% with a peak systolic pressure gradient across the stenosis of >20 mmHg undergoing PTA + Stenting* (*n* = 52; 93%)	
*Patients undergoing diagnostic angiography for suspicion of TRAS during follow-up* (*n* = 8, 14%)	

*Patients with re-TRAS treated with re-PTA + stenting* (*n* = 7)	*Patients with no evidence of TRAS continuing the regular follow-up protocol* (*n* = 1)

**Table 4:** *Comparison of SPV, RI, SBP, DBP, and eGFR values among the TRAS-patients before and after stenting placement*. TRAS = transplant renal artery stenosis; SPV = systolic peak velocity; RI = resistive indexes.

	Prestenting (*n* = 52)	Poststenting (*n* = 52)	*p* value
*Systolic peak velocity (SPV) at the level of TRAS (m/sec)* (median, IQR)	3,0 (2,6–3,6)	1,4 (1,2–1,7)	<0,001

*Resistive index (RI) at parenchymal level (n)* (median, IQR)	0,68 (0,62–0,73)	0,72 (0,69–0,77)	0,01

*Systolic blood pressure (mmHg)* (median, IQR)	145 (140–160)	140 (120–150)	0,1

*Diastolic blood pressure (mmHg)* (median, IQR)	85 (80–90)	80 (75–85)	0,06

*eGFR (ml/min/m2)* (median, IQR)	49 (35–56)	53 (41–63)	0,11

**Table 5:** *Comparison of ΔSPV, ΔRI, ΔSBP, ΔDBP, and ΔeGFR values among the TRAS-patients at different time periods from renal transplantation*. TRAS = transplant renal artery stenosis; SPV = systolic peak velocity; RI = resistive indexes.

	Time to TRAS treatment			*p* value
	<3 months (*n* = 16)	3–12 months (*n* = 11)	>12 months (*n* = 25)	
*Δ systolic peak velocity (SPV) at the level of TRAS (poststent SPV − prestent SPV)* (ml/min) (median, IQR)	−1,5 (−1,7; −0,7)	−1,6 (−1,9 −1,1)	−1,5 (−1,8; −1,2)	0,9

*Δ resistive index (RI) at parenchymal level (poststent RI − prestent RI)* (*n*) (median, IQR)	0,0 (−0,01; 0,09)	0,06 (0,00–0,12)	0,3 (0,01–0,08)	0,5

*Δ mean systolic blood pressure (SBP) (poststent SBP − prestent SBP)* (mmHg) (median, IQR)	−10,0 (−20,0; 0,0)	−10,0 (−40,0; 0,0)	−12,0 (−34; 2,0)	0,4

*Δ mean diastolic blood pressure (DBP) (poststent DBP − prestent DBP)* (mmHg) (median, IQR)	0,0 (−10; 5,0)	−10,0 (−20,0; 5,0)	5,0 (−14; 8,0)	0,3

*Δ mean eGFR (poststent − prestent) (ml/min/m2)* (median, IQR)	8,5 (−1,0; 25)	5,0 (−8,0; 16,0)	6,5 (−2; 18)	0,4

## Supplementary Materials

Supplementary Materials Supplementary Figure 1: flowchart depicting the follow-up results in our study cohort. Supplementary Table 1: clinical characteristics and functional outcomes at the latest follow-up among patients undergoing PTA + stenting in our study cohort. Of 52 patients, 9 (18%) patients were lost at follow-up.
