# Supplementary material for: Standardized Duplex Ultrasound-Based Protocol for Early Diagnosis of Transplant Renal Artery Stenosis: Results of a Single-Institution Retrospective Cohort Study
Source: Biomed Res Int. 2018 Apr 11;2018:2580181. doi: 10.1155/2018/2580181 (PMC5925009; doi:10.1155/2018/2580181)

Supplementary Table 1. Clinical characteristics and functional outcomes at the latest follow-up among patients undergoing PTA + stenting in our study cohort. Of 52 patients, 9 (18%) patients were lost at follow-up.

**Patients alive at follow-up**							
At the time of surgery					***At last follow-up***		
**Patient n.**	**Gender**	**Cause of CKD**	**Age-adjusted Charlson Comorbidity Index (excluding CKD)**	**BMI**	**eGFR (CKD-EPI 2009, ml/min)**	**Re-Dialysis**	**Graft survival (mo)**
1	Male	Fabry disease	2	29,4	44	No	128
2	Female	Chronic Pyelonephritis	1	28,1	59	No	166
3	Male	Chronic Pyelonephritis	1	20,2	40	No	78
4	Male	ADPKD	1	25,7	45	No	62
5	Male	Chronic Pyelonephritis	2	22,9	73	No	150
6	Male	ADPKD	1	23,0	82	No	77
7	Female	Chronic Glomerulonephritis	3	24,5	<15	Yes	129
8	Male	ADPKD	2	24,7	65	No	55
9	Female	ADPKD	1	30,8	46	No	167
10	Female	Chronic Glomerulonephritis	3	24,0	37	No	187
11	Female	ADPKD	2	26,0	34	No	155
12	Male	Ureteral Anomalies	3	26,0	<15	Yes	64
13	Male	Hypertension	3	25,5	21	Yes	113
14	Female	Chronic Glomerulonephritis	2	20,9	27	No	154
15	Male	ADPKD	1	26,6	39	No	204
16	Male	Vescicoureteral reflux	1	31,8	30	No	60
17	Female	Chronic Glomerulonephritis	4	18,7	31	No	131
18	Male	ADPKD	3	21,3	38	No	98

19	Female	Chronic Glomerulonephritis	2	26,5	56	No	128
20	Female	ADPKD	2	24,9	43	No	56
21	Male	ADPKD	2	26,6	25	No	72
22	Male	Chronic Glomerulonephritis	3	22,5	19	Yes	130
23	Female	Hypertension	4	21,8	<15	Yes	86
24	Male	Chronic Glomerulonephritis	3	27,2	18	Yes	222
25	Female	LES	4	29,0	<15	Yes	84
**Patients dead at follow-up**							
At the time of surgery					***At last follow-up***		
**Patient n.**	**Gender**	**Cause of CKD**	**Age-adjusted Charlson Comorbidity Index (excluding CKD)**	**BMI**	**Cause of death**	**Re-Dialysis**	**Graft survival (mo) *at death***
1	Male	Nephroangiosclerosis	3	23,5	Kaposi Sarcoma	No	24
2	Female	Nephronophtisis	4	21,7	CMV infection	No	8
3	Male	Iatrogenic Interstitial Nephritis	2	22,0	Unknown	No	147
4	Male	Chronic Glomerulonephritis	2	27,0	Sepsis	No	110
5	Male	Chronic Glomerulonephritis	2	26,6	Skin Cancer	No	58
6	Male	Chronic Pyelonephritis	1	20,0	Respiratory Failure	No	191
7	Male	Unknown	3	24,4	Stroke	No	35
8	Male	Chronic Glomerulonephritis	3	25,2	Prostate Cancer	No	46
9	Female	Chronic Glomerulonephritis	3	24,1	Stroke	No	153

10	Male	ADPKD	3	25,6	Cerebral infection	No	103
11	Male	Chronic Glomerulonephritis	3	27,8	Skin Cancer	No	285
12	Female	LES	4	28,2	Bladder Cancer	Yes	56
13	Male	ADPKD	2	24,4	Stroke	No	18
14	Female	Chronic Pyelonephritis	2	22,8	Sepsis	Yes	58
15	Male	LES	2	21,9	HSV infection	No	65
16	Male	Chronic Glomerulonephritis	2	22,8	Skin Cancer	No	97
17	Female	Chronic Glomerulonephritis	3	25,8	Myocardical infarction	Yes	119
18	Male	LES	2	28,1	CMV infection	No	87

Supplementary Figure 1. Flow-chart depicting the follow-up results in our study cohort.

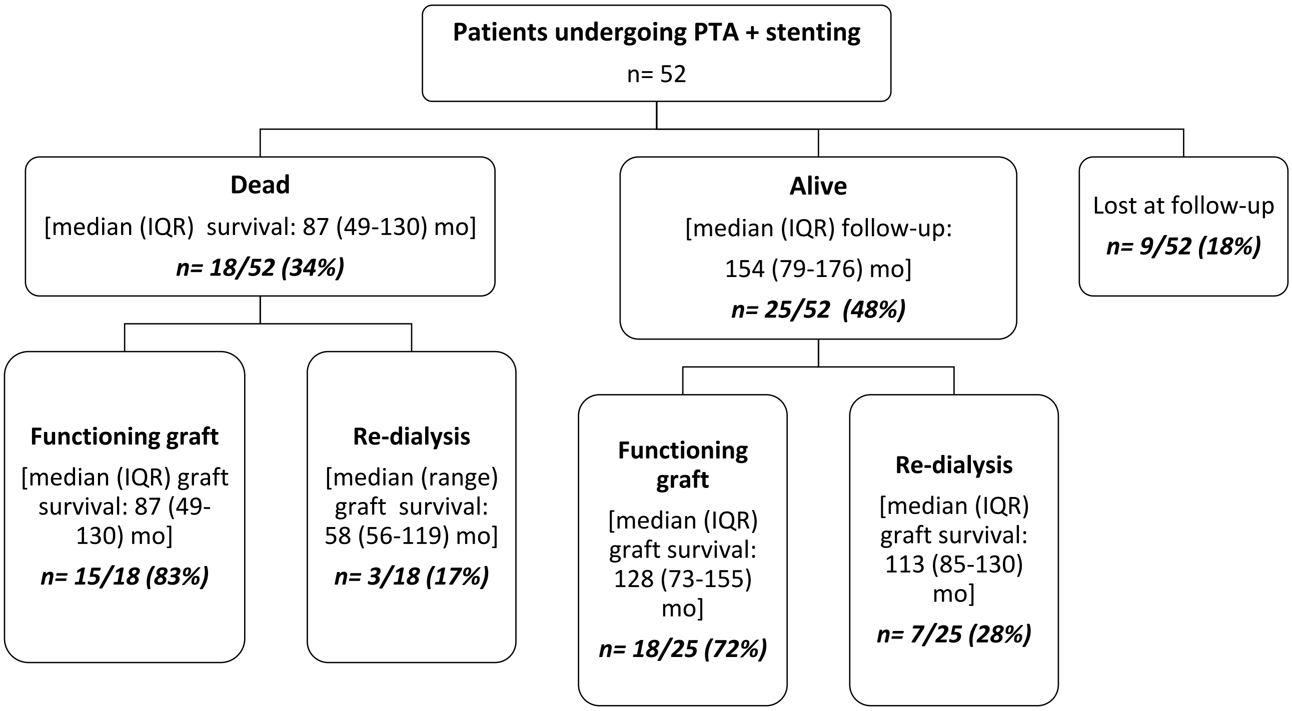

Supplement: Supplementary Materials — Supplementary Figure 1: flowchart depicting the follow-up results in our study cohort. Supplementary Table 1: clinical characteristics and functional outcomes at the latest follow-up among patients undergoing PTA + stenting in our study cohort. Of 52 patients, 9 (18%) patients were lost at follow-up. [file 2580181.f1.pdf]
